# The mechanism of dietary cholesterol effects on lipids metabolism in rats

**DOI:** 10.1186/1476-511X-9-4

**Published:** 2010-01-14

**Authors:** Yu-Ming Wang, Bei Zhang, Yong Xue, Zhao-Jie Li, Jing-Feng Wang, Chang-Hu Xue, Teruyoshi Yanagita

**Affiliations:** 1 College of Food Science and Engineering, Ocean University of China, Qingdao, China; 2 Department of Applied Biological Sciences, Saga University, Saga, Japan

## Abstract

**Background:**

Cholesterol administration has been reported to influence hepatic lipid metabolism in rats. In the present study, the effect of dietary cholesterol on hepatic activity and mRNA expression of the enzymes involved in lipid metabolism were investigated. Fourteen male Wistar rats were randomly divided into 2 groups and fed 1% cholesterol or cholesterol free AIN76 diets for 4 weeks.

**Results:**

The serum triglyceride and high density lipoprotein cholesterol levels were significantly decreased but the total cholesterol and non high density lipoprotein cholesterol levels were significantly increased in the cholesterol-fed rats compared with the control rats. And the concentrations of the hepatic total cholesterol and triglyceride increased about 4-fold and 20-fold separately by dietary cholesterol. The activities of hepatic malic enzyme, glucose-6-phosphate dehydrogenase, fatty acid synthase, phosphatidate phophatase and carnitine palmitoyl transferase were depressed by the cholesterol feeding (40%, 70%, 50%, 15% and 25% respectively). The results of mRNA expression showed that fatty acid synthase, carnitine palmitoyl transferase 1, carnitine palmitoyl transferase 2, and HMG-CoA reductase were down-regulated (35%, 30%, 50% and 25% respectively) and acyl-CoA: cholesterol acyltransferase and cholesterol 7α-hydroxylase were up regulated (1.6 and 6.5 folds) in liver by the cholesterol administration.

**Conclusions:**

The dietary cholesterol increased the triglyceride accumulation in liver, but did not stimulate the activity and the gene expression of hepatic enzymes related to triglyceride and fatty acid biosynthesis.

## Background

The high dietary cholesterol was concerned with the increasing concentrations of serum and hepatic total cholesterol(TC), especially the level of very low density lipoprotein (VLDL) and low density lipoprotein (LDL) in serum, which is considered to be a primary risk factor of cardiovascular disease. Hypercholesterolemia rat model is represented for cardiovascular and cerebrovascular disease research, which can be established by feeding with 0.5%-1.0% cholesterol-supplement diet for several weeks. Dietary 0.5%-1.0% cholesterol can increase serum VLDL and LDL levels dramatically in rats. In this case, dietary cholesterol remarkably disturbed triglyceride (TG) metabolism, the hepatic TG content increased in folds until hepatic steatosis forms. Thereby, it has been developed as a non-alcoholic fatty liver disease (NAFLD) model induced by diet in some previous studies. However, the mechanism of the rat hypercholesterolemia and NAFLD by high dietary cholesterol has not been systematically investigated.

Numerous studies have been done to seek the effect of dietary cholesterol on hepatic lipid homeostasis. In early 1990s, Thomas *et al. *[1,2] has investigated the effect of cholesterol on the accumulation of liver lipids through the radioisotope ^14^C-fatty acid and proposed that the hepatic TG accumulation was developed by the enhancement of hepatic TG synthesis and the reduction of fatty acid beta-oxidation. Liu *et al*. [3] has suggested the roles of increased lipogenesis, decreased oxidation of fatty acids and decreased secretion of VLDL as causes for the accumulation of TG in the liver in the cholesterol-fed rats. Xu *et al*. [4] has also reported that the impaired hepatic lipid homeostasis because of lipid accumulation attributed to the increasing activity of the enzymes involved in fatty acid biosynthesis in the rats by the dietary cholesterol. But in our study, we found that the activity of the enzymes correlated to lipogensis capacity did not increase by the dietary cholesterol. Besides, some researchers [5] thought the cholesterol synthesis was abolished in the high-cholesterol-fed rats, while we suggested the cholesterol administration affected not only the cholesterol catabolism but also the cholesterol biosynthesis. So the mechanism of dietary cholesterol on lipid metabolism is still unclear.

As we know, VLDL is assembled in liver and secreted to serum, and high hepatic TG level induces high serum TG concentration. That is because VLDL is mainly composed of TG. But our previous research found that cholesterol feeding significantly increased the hepatic TG level, but reduced the serum TG content obviously as compared with the controls. So we came up with our hypothesis based on the above that incorporation of cholesterol ester (CE) into the VLDL particles takes priority amid VLDL assembly in comparison with TG in cholesterol-fed rats. In order to test and verify our hypothesis, we focused on the effect of dietary cholesterol on the activity of the enzymes involved in fatty acid and cholesterol metabolism in rat liver. The enzymes correlated to TG homeostasis were determined as follows: fatty acid synthase(FAS), malic enzyme(ME) and glucose-6-phosphate dehydrogenase (G6PDH), and carnitine palmitoyl transferase (CPT). To gain intensive insight into fatty acid metabolism, the following genes expressing the following proteins were measured: CPT1 and CPT2. With regard to the cholesterol homeostasis, the activity of phosphatidate phophatase (PAP) and the mRNA expression of HMG-CoA reductase, acyl-CoA: cholesterol acyltransferase (ACAT) and cholesterol 7α-hydroxylase1 (CYP7A1) were measured.

## Materials and methods

### *Animals and diets*

Fourteen male Wistar rats, 5 wk old, were purchased from Qingdao animal center (Qingdao, China). The rats were housed in metal cages in a temperature-controlled room under a 12-h light/dark cycle. After a 1 wk adaptation period on powder chow diet, the rats were randomly assigned to 2 groups (n = 7) according to the fasted body weights and pair-fed (providing the rats each day with the amount of food eaten by matched freely fed animals) with 1% cholesterol or cholesterol free AIN76 diet (Table 1). After 4 wk feeding, the final fasted body weight was weighed, and all rats were killed by aortic exsanguination under diethyl ether anesthesia. Liver and white adipose tissues(W.A.T.s, perirenal, epididymal, omental and subcutaneous fat) were excised immediately, and serum was separated from the blood for analysis. All aspects of the experiment were conducted according to the guidelines provided by the ethical committee of experimental animal care at Ocean University of China (OUC, China).

**Table 1:** Compositions of diets for different rat groups

	Control	Cholesterol
Casein(g)	20	20
Starch(g)	15	15
Vitamin(AIN-76)(g)	1	1
Mineral(AIN-76)(g)	3.5	3.5
Choline bitartrate(g)	0.2	0.2
Cellulose(g)	5	5
DL-Methionine(g)	0.3	0.3
Corn oil(g)	10	10
Sucrose(g)	45	43.75
Cholesterol(g)	0	1
Bile salt (pig)(g)	0	0.25
Total(g)	100	100

### Serum and liver lipids

Serum was separated by centrifuging the blood at 7500*g for 15 min. Triglyceride (TG, Cat.F001), total cholesterol (TC, Cat.F002), high density lipoprotein-cholesterol (HDL-c, Cat.F003), non-esterified fatty acid (NEFA, Cat.A042), and glucose(Cat.F006) were measured using enzymatic reagent kits from Biosino (Beijing, China). Phospholipid (PL, Cat. 467-32101) was measured using assay kit from Wako Pure Chemicals(Tokyo, Japan). Liver lipids were extracted and purified according to the method of Folch *et al*. [6]. The concentrations of TG, TC and PL were measured according to the methods of Fletcher [7], Sperry [8] and Bartlett [9].

### Assays of enzyme activity

A piece of liver was homogenized in a 0.25 mol/L sucrose solution that contained 1 mol/L of ethylene-diaminetetraacetic diaminetetraacetic acid (EDTA) in a 10 mol/L Tris-HCl buffer (pH 7.4). After precipitating the nuclei fraction, the supernatant was centrifuged at 10,000*g for 10 min at 4°C to obtain mitochondria. The resulting supernatant was recentrifuged at 125,000*g for 60 min to precipitate microsomes, and the remaining supernatant was used as the cytosol fraction. The protein concentration was determined according to the method of Lowry *et al.*, with bovine serum albumin used as the standard. The enzyme activities of ME, G6PDH and FAS[10-12] in the liver cytosol fraction, mitochondrial CPT[11] and microsomal PAP [13] were determined as described.

### Determination of mRNA levels from liver

Total cellular RNA was isolated from liver samples using the Trizol reagent (Invitrogen, Caelsbad, CA), according to the manufacturer's recommended procedures. Assays-on-Demand, Gene Expression Products [Mm00662319_m1 for FAS, Mm00486279_m1 for ACAT, and Hs99999901_s1 for 18SRNA, Applied Biosystems, Tokyo, Japan) and TaqMan MGB Gene Expression Kits for HMG-CoA reductase (forward primer,5'-AGTGATTGTGTCAGTATTATTGTGGAAG-3';reverse primer,5'-GGTA CTGGCTGAAAAGTCACAAGAG-3'; and TaqMan MGB probe, 5'-FAM-TTGCTGTTGTATGTAAAG\T-MGB-3') were used for the quantitative real-time reverse transcribed polymerase chain reaction(RT-PCR) analysis of FAS, ACAT, 18SRNA, and HMG-CoA reductase expression in the liver. The amplification was performed with a real-time PCR system (ABI Prism 7000 Sequence Detection System; Applied Biosystems). Results were expressed as a relative value after normalization to18S RNA[14].

### Statistical analysis

All values are expressed as mean ± S.E.M. Means of the two groups were compared by *Student's t test *for each experiment. Differences were considered to be significant at *P *< 0.05.

## Results

### Effects of cholesterol on the growth parameters

Table 2 summarized the growth parameters of Wistar rats after 4 weeks of feeding on the different diets. Though there was no significant difference in body weight gain, food intake or total white adipose tissues between the two groups, the cholesterol diet drastically increased the liver weight (*P *< 0.001).

**Table 2:** Effects of cholesterol on the growth parameters in Wistar rats^1^

	Control	Cholesterol
Initial B.W. (g)	197 ± 2	197 ± 2
Final B.W. (g)	305 ± 4	310 ± 6
Food intake (g/d)	24.8 ± 0.5	24.6 ± 0.4
Total W.A.T. (g/100 g B.W.)	5.62 ± 0.38	4.99 ± 0.34
Liver weight (g)	12.1 ± 0.7	17.0 ± 0.9*

^1^Data are presented as mean ± S.E.M. n = 7 rats per group.

B.W., body weight; W.A.T., white adipose tissue.

*P < 0.001, Compared to control group.

### Effects of cholesterol on the concentrations of serum lipids and glucose

As shown in Table 3, the concentrations of the serum TC and non-HDL-c were significantly higher (*P *< 0.001, *P *< 0.001) but the HDL-c and TG levels were remarkably lower (*P *< 0.001, *P *< 0.001) *i*n the cholesterol-fed rats compared with the control rats. These results indicated that the cholesterol administration developed lipid metabolic dysfunction, and promoted hyperlipidemia. Additionally, the cholesterol treatment did not affect the serum PL, NEFA and glucose levels. The concentration of serum phospholipids, the main contents of the surface of lipoprotein, did not significantly change by dietary cholesterol, but the LDL-c level was dramatically higher (*P *< 0.001) in the cholesterol group than controls. The result may illustrate that the serum lipoprotein of the cholesterol-fed rats mainly consist of LDL of large particle size, which was abundant in cholesterol, or dietary cholesterol suppressed the TG level and increased the concentration of CE in the serum lipoprotein.

**Table 3:** Effects of cholesterol on concentrations of serum lipids and glucose in Wistar rats^1^

	Control	Cholesterol
TG (mmol/L)	1.27 ± 0.16	0.67 ± 0.11*
TC (mmol/L)	2.29 ± 0.18	4.92 ± 0.91*
HDL-c (mmol/L)	1.57 ± 0.09	0.78 ± 0.07*
(VLDL+LDL)-c (mmol/L)	0.72 ± 0.17	4.14 ± 0.96*
PL(mmol/L)	2.08 ± 0.13	1.91 ± 0.05
NEFA (mmol/L)	0.453 ± 0.037	0.420 ± 0.039
Glucose (mmol/L)	9.78 ± 0.61	9.33 ± 0.44

^1^Data are presented as mean ± S.E.M. n = 7 rats per group.

TG, triglyceride; TC, total cholesterol; HDL-c, high density lipoprotein cholesterol; VLDL-c, very low density lipoprotein cholesterol; PL, phospholipid; NEFA, non-esterified fatty acid.

*P < 0.001, compared to control group.

### Effect of cholesterol on the concentrations of hepatic lipid

Table 4 showed the hepatic lipid levels of Wistar rats after 4 weeks of feeding of the diets. Cholesterol feeding disturbed hepatic lipid metabolism in rats. The concentrations hepatic TC and TG remarkably increased by 19.2 folds (*P *< 0.001) and 2.52 folds (*P *< 0.001) respectively, while the PL level decreased significantly (15.4%, *P *< 0.001) in cholesterol group as compared with the controls.

**Table 4:** Effects of cholesterol on concentrations of hepatic lipid in Wistar rats^1^

	Control	Cholesterol
TG (mmol/100 g Liver)	4.23 ± 0.80	10.6 ± 1.02*
TC (mmol/100 g Liver)	0.93 ± 0.05	17.93 ± 0.57*
PL (mmol/100 g Liver)	4.61 ± 0.12	3.90 ± 0.12*

^1^Data are presented as mean ± S.E.M. n = 7 rats per group.

TG, triglyceride; TC, total cholesterol; PL, phospholipid.

*P < 0.001, compared to control group.

### Effect of cholesterol on enzyme activity involved in hepatic lipid metabolism

Table 5 demonstrated that cholesterol administration significantly inhibited the activities of the enzymes related to lipogenesis such as FAS, ME and G6PDH, and the activity of PAP, a rate-limiting enzyme in TG synthesis by 50% (*P *< 0.001), 40% (*P *< 0.001), 70% (*P *< 0.001) and 15% (*P *< 0.001 )respectively as compared with the control diet. Moreover, the hepatic mitochondrial beta-oxidation, whose rate-limiting enzyme is CPT was remarkably suppressed by 25% (*P *< 0.001 ) by dietary cholesterol. Thus, the cholesterol administration reduced the activity of hepatic enzymes involved in fatty acid biosynthesis and beta-oxidation and TG synthesis.

**Table 5:** Effects of cholesterol on enzyme activities involved in hepatic lipid metabolism^1^

	Control	Cholesterol
ME(nmol/min·mg protein )	47.6 ± 4.0	27.7 ± 1.6 *
G6PDH(nmol/min·mg protein)	64.8 ± 7.5	18.7 ± 1.2*
FAS(nmol/min·mg protein )	9.71 ± 1.64	5.19 ± 0.86*
PAP(nmol/min·mg protein )	21.2 ± 1.0	18.2 ± 1.1**
CPT(nmol/min·mg protein )	6.54 ± 0.40	5.00 ± 0.37**

^1^Data are presented as mean ± S.E.M. n = 7 rats per group.

ME, malic enzyme; G6PDH, glucose-6-phosphate dehydrogenase; FAS, fatty acid synthase; PAP, phosphatidate phophatase; CPT, carnitine palmitoyl transferase

*P < 0.05,**P < 0.01, compared to control group.

### Effect of cholesterol on mRNA expression of hepatic fatty acid metabolism enzymes

**Figure 1:**
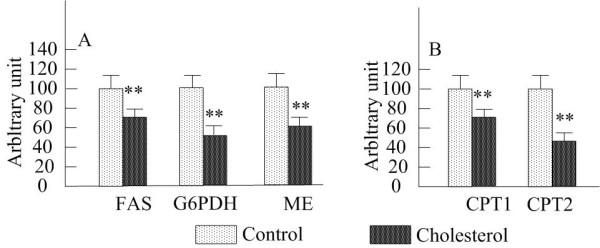
**Effects of cholesterol on mRNA expression of hepatic fatty acid metabolism enzymes in rats**. n = 7 rats per group. Data are presented as mean ± S.E.M. FAS, fatty acid synthase; G6PDH, glucose-6-phosphate dehydrogenase; CPT, carnitine palmitoyl transferase.*P < 0.05,**P < 0.01, compared to control group.

Figure 1 showed hepatic mRNA levels of Wistar rats after 4 weeks of feeding of the diets. Consistent with the activity of the enzymes correlated to lipogenesis capacity, mRNA expression of FAS, G6PDH and ME decreased (35%,59% and 44%) (*P *< 0.01)dramatically by dietary cholesterol (Figure 1A). As shown in Figure 1B, the cholesterol administration significantly depressed the mRNA abundances of CPT1 (*P *< 0.01) and CPT2 (*P *< 0.01), which corresponded with the change of the activity involved in fatty acid beta-oxidation.

### Effect of cholesterol on mRNA expression of hepatic cholesterol metabolism enzymes

**Figure 2:**
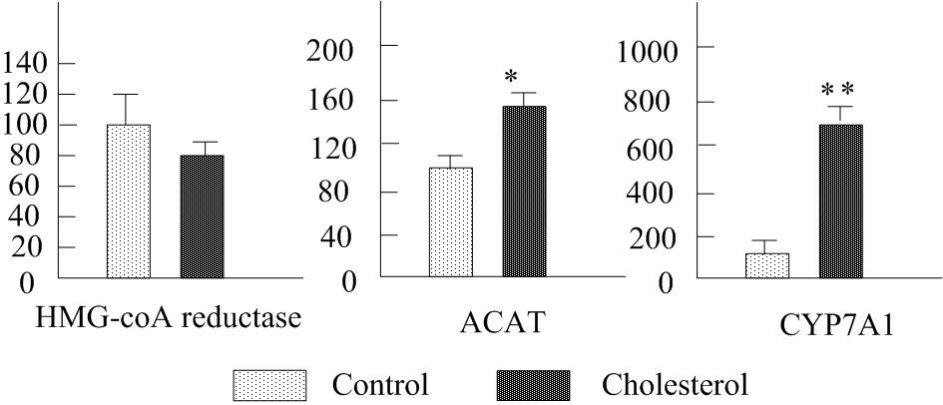
**Effects of cholesterol on mRNA expression of hepatic cholesterol metabolism enzymes in rats**. n = 7 rats per group. Data are presented as mean ± S.E.M. ACAT, acyl-CoA: cholesterol acyltransferase; CYP7A1, cholesterol 7α-hydroxylase1.*P < 0.05,**P < 0.01, compared to control group.

Figure 2 showed the hepatic mRNA abundances of Wistar rats after 4 weeks of feeding of the diets. HMG-CoA reductase is the rate-controlling enzyme of cholesterol biosynthesis pathway. The mRNA level of HMG-CoA reductase decreased by 25%, however without statistical significance in the cholesterol-fed rats in comparison with the control-diet rats. ACAT, a rate-controlling enzyme of cholesterol esterification, and CYP7A1, a rate-limiting enzyme of cholesterol hydroxylation, relate hepatic cholesterol catabolism. mRNA levels of the two enzymes were increased significantly in the cholesterol group as compared with the controls. The result presented here showed the enhancement of cholesterol hydroxylation and esterification and the suppression of hepatic cholesterol synthesis. An interpretation of this fact documented that the redundant cholesterol was expelled from the liver.

## Discussion

This study assessed the mechanism of the effect of dietary cholesterol on lipid metabolism in rats. The results demonstrated that the cholesterol administration significantly increased the concentrations of the hepatic TC and TG in rats (Table 4), which indicated that dietary cholesterol obviously disturbed hepatic lipid metabolism. Hepatic TG level is controlled mainly by TG synthesis, beta-oxidation and secretion in form of lipoprotein [15]. Thomas et *al. *has reported that the hepatic TG accumulation by high dietary cholesterol was involved in the stimulation of fatty acid and TG synthesis in rats. But there existed other studies indicating that the cholesterol diet did not motivate the hepatic TG homeostasis [16,17]. In the present study, the enzyme activity and mRNA expression related to fatty acid and TG synthesis were measured to investigate the effect of dietary cholesterol on the distribution of TG and fatty acid to the liver. In the result, the activities of ME, G6PDH and FAS responsible for fatty acid synthesis in cholesterol-fed rats were significantly lower than those in controls (Table 5). And so it was with the activity of PAP, a rate-limiting enzyme in TG biosynthesis. The down-regulation of the genes was presented to coincide with the reduction of the activity of the enzymes in the cholesterol-fed rats (Figure 1A). Thus, we supposed that the hepatic TG accumulation by dietary cholesterol was not induced by the enhancement of the activity of the enzymes involved in fatty acid and TG synthesis.

On the other hand, hepatic fatty acid beta-oxidation is another factor to affect the hepatic fatty acid level. CPT1 is the rate-limiting enzyme of the mitochondria α-oxidation pathway. In the present study, hepatic CPT1 activity (Table 5) and CPT1a and CPT2 mRNA levels (Figure 1B) were inhibited drastically by the cholesterol treatment. Those results demonstrated that the cholesterol administration reduced fatty acid catabolism, which may account for the enhancement of hepatic TG level. Moreover, Fukada N *et al. *[18] have shown that the suppression of fatty acid beta-oxidation may be responsible for the conversion of fatty acid to TG and CE. That was probably another reason to illustrate the escalation of hepatic TG level.

Here we found that the serum TG level was significantly lower in the cholesterol-fed rats than controls. Whereas, other reports showed that hypercholesterolemia rats induced by high cholesterol diet had higher serum TG level. Thomas *et al.*[1,2] observed that cholesterol feeding increased the serum TG level in rats fed with low fat diet (5%), but had no effect on the concentration of serum TG in rats fed with high fat diet (20%). Pang *et al. *[19] reported that cholesterol treatment reduced the serum TG level in rats fed with high cholesterol diet. Van. *et al*.[20] showed that the kind of dietary fat was important for the serum TG level effected by dietary cholesterol. Hence, there were indications that the effects of cholesterol on serum TG level may be different depending on the distinct animal metabolism states and diet compositions.

Besides, as we know, phospholipids are the main contents of the surface of lipoprotein, and TG and CE inside of it. This study showed that the cholesterol feeding increased the serum LDL-c by 6-fold, and decreased the serum TG level by 50%, but had no effect on PL (Table 3). This fact may illustrate that the serum lipoprotein was mainly consist of LDL abundant in CE in the cholesterol-fed rats. Hence, we hypothesized that the CE preferentially flew into the inside of LDL particle, as compared with the TG particle, amid the LDL synthesis and secretion in the rats fed with cholesterol.

There are two pathways to reduce the hepatic cholesterol level: cholesterol catabolism or incorporation into the lipoprotein particles and release to the plasma in the form of CE. Figure 2 presented that the gene expression of HMG-CoA reductase, the key enzyme of cholesterol synthesis, suppressed significantly in the cholesterol-fed rats as compared with the control rats, which suggested that the hepatic cholesterol biosynthesis was inhibited by the high dietary cholesterol. As to the cholesterol degradation, the liver-specific enzyme CYP7A1, serves as a rate-limiting enzyme in the classical pathway of bile acid conversion into cholesterol, which is necessary for elimination of cholesterol excess [21]. The up-regulation of CYP7A1 mRNA indicated the increasing clearance of circulating cholesterol out of liver by dietary cholesterol. Hepatic cholesterol integrates into the lipoprotein particles to form CE, which plays the role of releasing cholesterol to plasma. While ACAT acts as a rate-controlling enzyme of cholesterol esterification, the enhancement of mRNA expression of ACAT demonstrated the increasing cholesterol catabolism by the cholesterol treatment (Figure 2). The result also confirmed our presumption that the serum lipoprotein consist of LDL, which CE preferentially flew into by the cholesterol administration.

## Conclusions

Our finding suggested that the hepatic TG accumulation caused by dietary cholesterol may attribute to the reduction of fatty acid beta-oxidation and the preference of CE to afflux to LDL during the onset of biosynthesis and secretion of LDL, not due to the enhancement of the activity of the enzymes related to the fatty acid and TG synthesis. But the detailed mechanism still needs further investigation.

## Competing interests

The authors declare that they have no competing interests.

## Authors' contributions

YMW contributed in design, experimental work, analysis and publication of results. BZ was responsible for drafting the manuscript. YX participated in the design of the study, animal studies and performed statistical analysis. ZJL was in charge of planning and discussing the results. JFW participated in the design of the study and statistical analysis. CHX participated in drafting the manuscript, discussion of results and providing funding for the study. TY participated in the design and provided funding for the study. All authors have read and approved this manuscript.
